# Quality and Processability of Modern Poultry Meat

**DOI:** 10.3390/ani12202766

**Published:** 2022-10-14

**Authors:** Shai Barbut, Emily M. Leishman

**Affiliations:** 1 Department of Food Science, University of Guelph, Guelph, ON N1G 2W1, Canada; 2 Department of Animal Biosciences, University of Guelph, Guelph, ON N1G 2W1, Canada

**Keywords:** broiler, color, myopathy, processing, safety, taste, turkey

## Abstract

**Simple Summary:**

Over the years, the poultry industry has evolved from selling mainly whole birds to mostly cut-up portions or further processed products. Additionally, changes in breeding and production have resulted in birds that grow bigger, faster, or produce more eggs for longer. To accommodate the changes, the industry has moved to more automation and increasing the number of birds that can be processed at once. However, this increase in efficiency and consumer desire for more convenient products (i.e., cut-up, ready-to-eat) comes with its own challenges since it is even more important that birds are more uniform/similar in their size, color, and texture. This is especially challenging given the rise in meat color, texture, and quality inconsistencies. This review focuses on the changes that occurred in poultry processing to date and describes challenges faced by modern poultry processors.

**Abstract:**

The poultry meat industry has gone through many changes. It moved from growing dual-purpose birds (meat and egg production) taking ~110 days to reach 1.2 kg 100 years ago, to developing specialized meat breeds that grow to 2.5 kg within ~40 days. It also moved from selling ~80% whole birds to mostly selling cut up and further processed products in the Western world. This necessitated building large, centralized processing plants, capable of processing 15,000 birds per hr on a single line (60 years ago only 2500), that require higher bird uniformity (size, color, texture). Furthermore, consumer demand for convenient products resulted in introducing many cut-up fresh poultry (some companies have 500 SKU) and further processed products (chicken nuggets did not exist 50 years ago). Those developments were possible due to advancements in genetics, nutrition, medicine, and engineering at the farm and processing plant levels. Challenges keep on coming and today a rise in myopathies (e.g., so called woody breast, white striping, spaghetti meat), requires solutions from breeders, farmers, and processing plants, as more automation also requires more uniformity. This review focuses on the changes and challenges to the processing industry segment required to keep supplying high quality poultry to the individual consumer.

## 1. Introduction

This review focuses on modern poultry production where over 80% of poultry is sold as cut-up parts or further processed products [1]. This is especially true in Western markets where 80 years ago >80% of the poultry was sold as whole birds (live or eviscerated) and only a small portion was cut up in small butcher shops [1]. This major change in marketing is the result of consumers having more income to spend on food and consumers looking for convenient semi/fully prepared food products (e.g., ready-to-eat fried chicken, and turkey pastrami). Demographic changes such as both parents working outside the home have also been a strong driving force in increasing the trend of buying prepared foods, and today people spend significantly less time on food preparation at home compared to 60 years ago (15 vs. 90 min, respectively) [1].

To meet these demands, the poultry industry built large, dedicated processing plants where line speed is continuously increasing. Today, a single fast broiler processing line can deal with 15,000 birds per hr. This is a 6-fold increase over line speed 60 years ago [1]. Increasing line speed has not been an easy task, and progress has been as a result of advancements in automation and mechanization of different processes within the plant [2]. Examples are the conversion of the early batch-type scalding and defeathering process into a continuous line. This was later followed by the introduction of automation into the evisceration and later also the cut-up and deboning lines. These developments have been geared to replace repetitive manual labor as well as increase line speed and efficiency [3]. This has obviously required a lot of work (a few hundred patents have been filled), and new challenges continue to appear periodically. A current challenge is the rise of myopathies such as the so-called woody-breast (hard connective tissue within the *Pectoralis major* muscle) and spaghetti meat (muscle bundle separating within parts of the *Pectoralis major* muscle); both are discussed below in greater detail. These myopathies are making existing automated, fast deboning challenging since the meat consistency is inhomogeneous. Another ongoing challenge is bird uniformity, in terms of size and weight, as this again is not conducive for automated equipment (e.g., within an average flock of 2.5 kg birds, some birds can weigh 3.0 while others 1.5 kg: a 100% difference in weight). Big plants that have more than one line are splitting the birds to separate lines adjusted to small, medium, and large birds to improve their efficiency [1].

**Figure 1:**
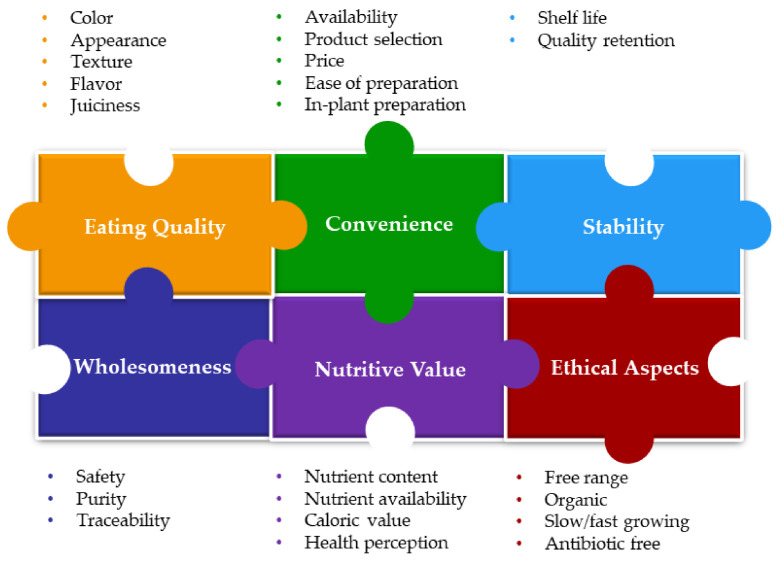
Example of interrelationships between factors that contribute to meat quality considering the perspectives of the producer, processor, and consumer. Note: as discussed in the text, the importance of the different factors depends on the industry segment.

The general topic of poultry meat quality has been discussed in several reviews which mostly focused on the eating quality of the final product, and factors affecting it such as breed, age of the bird, nutrition, and husbandry [3,4,5]. However, the term quality can have a different meaning depending on the user. For example, the farmer is interested in fast-growing, healthy birds, and a good feed conversion ratio. The processor is interested in uniformity (see discussion below about automation where uniformity is very important), high meat yield, and lack of defects (bruises, broken bones) [3]. The consumer, on the other hand, is focusing on factors such as texture, flavor, juiciness, and appearance [1,3,4,5]. This can be related to the definition of quality: “quality is the totality of features and characteristics of a product or service that bear on its ability to satisfy given needs” [6]. In contrast to previous reviews, this one focuses on the challenges facing the processor/meat processing industry in delivering high-quality poultry meat. In any case, when talking about products, one should also discuss price, as there are different quality expectations from a product at a different price range. Figure 1 illustrates the interrelations of the many parameters that contribute to quality and the main topics (listed in the colored shapes) will be discussed in greater detail below.

## 2. Uniformity and Processability

**Figure 2:**
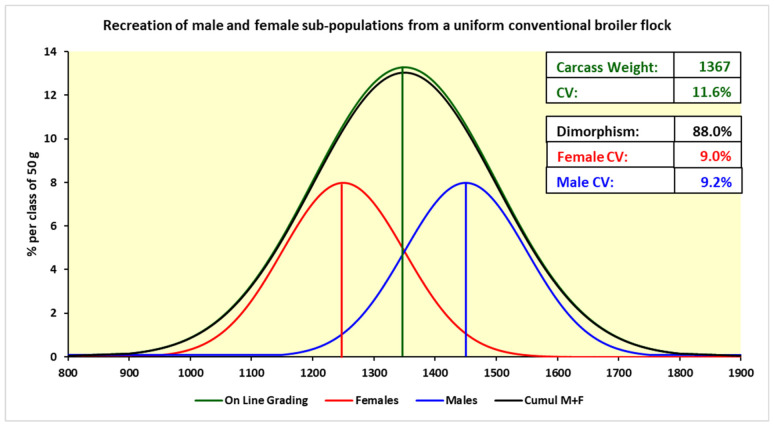
Sub-populations of males and females taken from as hatched conventional broiler flocks, redrawn to show the characteristics of on line weighing. Recreated from Toudic [9] with permission.

**Figure 3:**
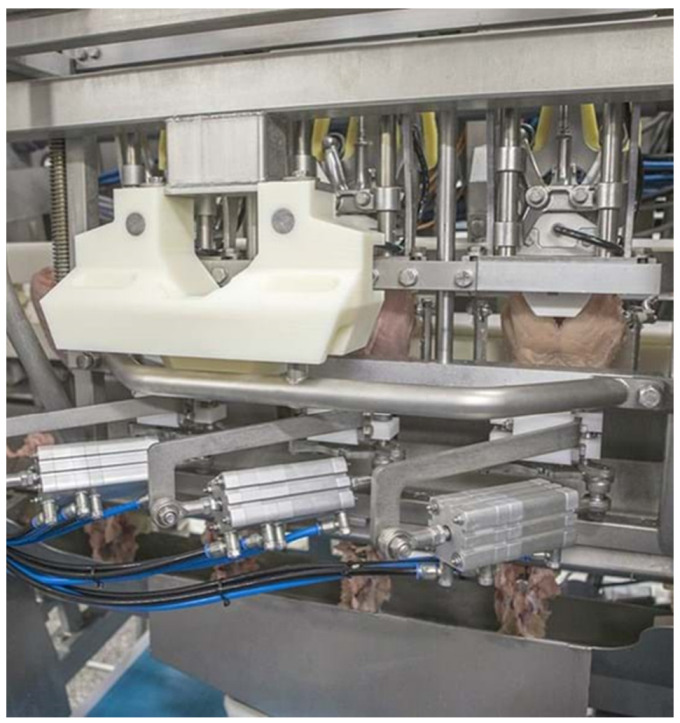
A new generation of automatic breast fillet deboning with the capabilities of measuring the size of each bone-in breast fillet portion to allow adaptive filleting of different size birds. The new generation machines also allow producing a range of products such as half fillets with/without skin, butterfly with/without tenders. Photo courtesy of Marel.

One of the key factors in introducing automation to processing is getting a good uniformity of specimens. This is not always easy when dealing with growing birds and the biological variations that exist within the flock. Figure 2 shows the distribution of the weight in a mixed flock of male and female broilers where the sex differences are shown. However, one should also pay attention to the range of weights within each sex. When it comes to automated processing one needs to adjust the equipment for optimal performance for a certain bird size (e.g., distance between the feather plucker’s fingers to remove feathers), and birds that are too small or too big can be either left with some feathers on or suffer from dislocated bones, respectively. When dealing with larger red-meat animals one can solve this problem by using scanning equipment (X-ray, ultrasound) to map the location of the bones and use a robot to perform a cutting operation [7]. However, one must realize that the line speed is much slower (5 to 10% of line speed for poultry) and the value of each carcass is much higher so it can justify the inclusion of a scanning step. It should be mentioned that some very new automated deboning machines used for high-value poultry cuts (e.g., breast fillets, thigh meat), do incorporate a measuring step (e.g., physical bone length, x-ray), so deboning of each individual part (e.g., breast cap, running at about 3500 per hour) can be adjusted and optimize to get a more efficient process. Figure 3 depicts a new generation of a breast fillet deboning machine with a sensor that can determine the size of each bone-in breast portion and adjust the cutting parameters. This development is helping to overcome some of the size uniformity challenges mentioned above, as adjusting the cutting setting improves yield and reduces the amount of meat staying on the bone (much lower value to the industry when processed as mechanically deboned meat). Another example of using sophisticated equipment to measure or scan parts is in the live animal side where equipment such as ultrasound and computer tomography (CT) are used to measure, in a non-destructive way, the size of different internal organs [8].

Today, various large poultry plants use automated weighing scales to separate the birds into light, medium, and heavy and then send them to three different lines which are adjusted accordingly. The industry is also looking for solutions from the breeding companies who can help by selecting certain birds for better size uniformity. Looking at the distribution in Figure 2, this can be described as the processors looking to narrow the distributions and also reduce the size of the two distribution tails. In any case, progress in this area will take quite some time.

It should also be noted that these differences in carcass weights determine portion sizes, which is very important in today’s market where supermarkets mainly demand fixed-weight packages. This challenge has been partially solved by automation where robots can pick and arrange products in a package with a fixed weight, and if needed also have a computer-controlled blade that cuts off some of the excessive meat (i.e., product giveaway can be very costly). It is also important to mention that there are breed effects with respect to uniformity and yield, where it was reported that a slow-growing breed showed higher yield but lower uniformity compared to two fast-growing breeds [10].

Increasing uniformity is also referring to other characteristics of the product, such as color, where the industry still has quite a variation within and between flocks as a result of challenges such as strain [11,12] and the so-called pale, soft and exudative (PSE) meat. The latter, as will be discussed in more detail under the ‘Color’ subheading below, is the result of some birds being more susceptible to stress (causing faster pH decline during rigor mortis) [13,14]. In any case, packing a very light-colored skinless breast meat fillet besides a darker one makes the consumer suspicious, and they are less likely to buy such a package, even though there is no safety/health concern with such an issue. It has been demonstrated that companies that sort the meat and make sure that all fillets in a package are of the same color have fewer products left on the shelf at the end of the day [15].

## 3. Eating and Appearance Qualities

### 3.1. Color

This is one of the key parameters in attracting/distracting consumers buying food in general and poultry is no exception. Some markets prefer yellow skin chickens while others prefer white color or some shades in between. This can be manipulated by the diet where feeding birds with feedstuffs containing carotenoids (e.g., corn base diet, spirulina) will result in pigment accumulation in the skin [16,17]. However, to maintain this color, the birds must be processed under mild scalding conditions so the pigment will not be rubbed off the product during the defeathering step [18]. An aggressive feather picking on one area of the carcass can also cause a patch of light color which results in downgrading [18]. Additional factors influencing poultry meat color have been reviewed elsewhere [5,14,16,19,20]. In brief, there are numerous factors which can play a role in poultry meat color including age (increasing haem pigment in the muscle), genotype (capacity for pigment fixation, fatness), muscle glycogen storage (glycolytic potential), and stress (exertion before slaughter).

**Figure 4:**
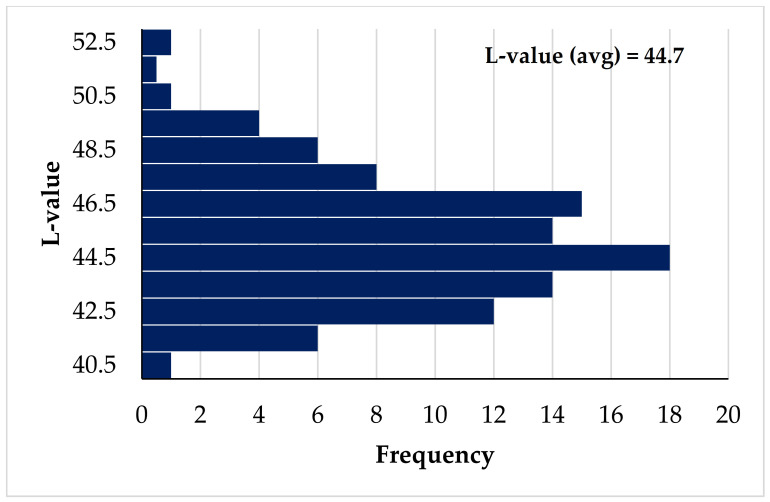
Redrawn histogram of L-value (lightness) distribution of breast meat fillets in young turkeys (*n* = 3000). Data from McCurdy et al. [21].

Overall, since most poultry sold in developed countries is already packed in some sort of plastic film (i.e., not allowing touching/smelling the meat), visual impression of color is extremely important. As mentioned above there can be quite some variation in the color of deboned poultry fillets. Figure 4 shows the color distribution in 3000 turkey breast fillets and demonstrates the existence of PSE and DFD (dark, firm dry) meat. As explained by McCurdy et al. [21], cases of DFD meat can be reduced if one understands that this is caused by muscle fatigue/depletion of the energy reserve (glycogen) that later is converted to lactic acid during the rigor mortis process (i.e., lower amount of glycogen results in higher post mortem pH, and darker color) [22]. Although PSE is generally thought to be the result of accelerated post mortem conversion of glycogen to lactic acid, reducing the occurrence of PSE in poultry is much more complicated and requires getting birds less susceptible to stress [13,23].

Discoloration due to bruises is also a negative factor and birds with a certain size bruise are downgraded (see additional info below). Bruises can typically occur prior to removing the birds from the farm or during transportation and tend to be found on the breast, thighs, and drums [24]. In some studies, it was reported, that the frequency of downgrades, in Canada, due to bruising on the drums alone was approximately 10% in turkeys and approximately 5% in broilers [25]. In Portugal, the incidence of bruises in broilers was reported to be 3.7% [26]. In Brazil, the prevalence of condemnations due to contusions, fractures, and bruising has been reported to range from 6–29% in a survey of two slaughterhouses [27]. The incidence of bruises at processing can be influenced by many factors. In particular, catching, transport, and slaughter conditions [28,29]. In general, longer distances, higher densities, and inappropriate electrical stunning tend to result in more bruises, as well as longer durations between shackling and slaughter [29]. It is interesting to mention that some studies today are looking at on-farm slaughtering to reduce/prevent some of these problems, including welfare issues [30].

### 3.2. Appearance

The way poultry is presented as parts or a whole bird has a significant effect on the purchasing decision of the consumer. Poultry that shows defects such as bruises, broken bones and/or discoloration are not going to sell, or they will sell at a substantial discount, since consumers associate physical defects with poor product quality [31]. This can be even if one wing is broken (<5% of a whole chicken weight). Therefore, processors try to avoid, as much as possible, releasing poultry with defects and trying to make sure such birds will go to cut up and sold as parts. The same is true if one sells a package of a dozen wings and one shows a big blood clot. It is also interesting to note that the grading systems in many countries are mainly focusing on aesthetics and lack of defects. For example, the Canadian system requires that whole birds showing a bruise or discoloration of more than 6.5 cm^2^ in the breast area or 8.0 cm^2^ elsewhere should go down from Grade A to Utility Grade [32]. Note that fairly similar values are listed in the US regulations [33].

In 1997, Barbut [34] already suggested replacing the grading system that focuses on many aesthetic factors with a system that takes into account actual meat quality parameters such as water holding capacity, gelation and emulsification capabilities; i.e., the document also suggested some numerical values for the new system. Overall, these parameters are much more important today, to both the processor and the consumer, when a significantly higher proportion of the meat is going to further processing and cut up. The latter is reflected by the fact that a number of large poultry processors have up to 500 stock keeping units (SKU) for fresh poultry portions, which can include items such as chicken breast meat ± skin, ± bone, ± marination, single/multiple fillets in a package/SKU.

Another interesting parameter in the current grading systems in most countries is the mandatory downgrading of poultry due to the presence of feathers on the carcass. According to the USA regulations, the presence of more than 4 feathers that are equal to or longer than a 1/2 inch cause the grade to go from A to B, while >6 feathers go to Grade C [33]. On the other hand, in some regions in Italy, consumers prefer to see one or two feathers on the broiler they buy.

### 3.3. Texture

**Figure 5:**
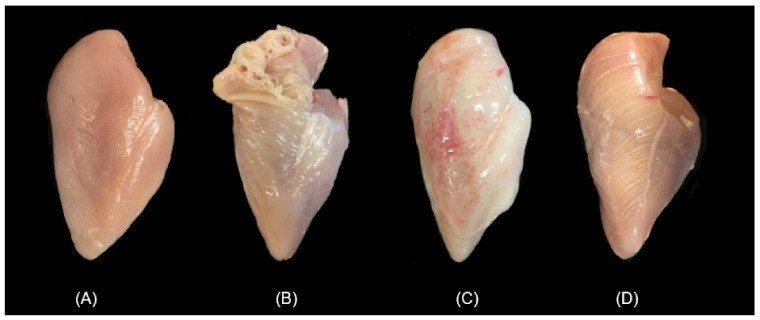
Myopathies in young broilers showing a normal fillet (**A**), spaghetti meat (**B**), the so-called woody-breast (**C**), and a fillet with white striping (**D**). Images by S. Barbut Lab.

**Figure 6:**
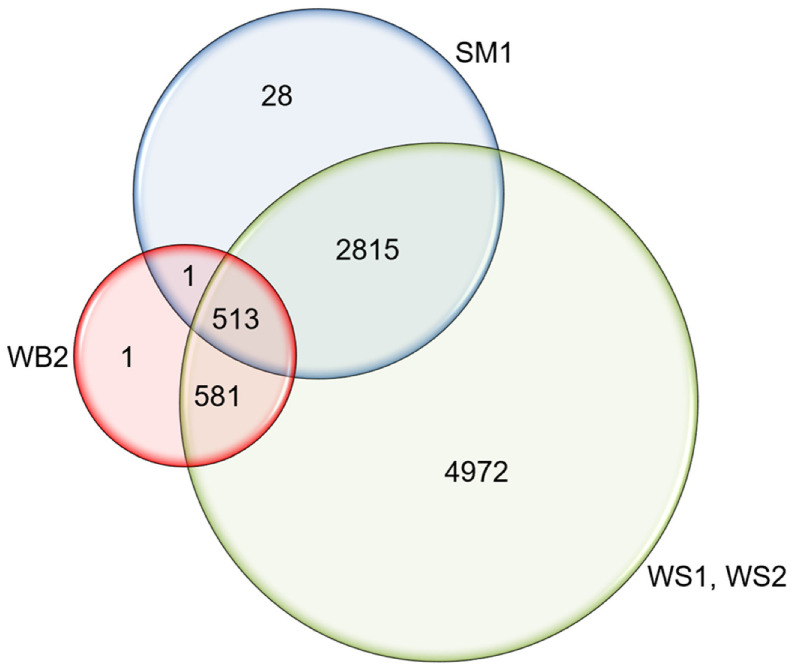
Venn Diagram showing the incidence counts of three myopathies in 8911 fillets. Spaghetti meat (SM) was observed in 3358 fillets, and severe woody breast (WB2) in 1096 fillets. Mild and moderate white striping (WS1 and WS2) were observed in 8881 fillets. The co-occurrence of SM and WB2 was observed in 514 fillets (Note: out of 9250 scored fillets, only 339 fillets did not show myopathies). Reproduced from Che et al. [37] with permission.

The texture of normal young poultry meat (broiler chickens 5–7 weeks of age; young turkey 14–20 months) in terms of toughness is usually not a problem unless there are glitches in the primary processing steps. Such problems can arise from early deboning and/or fast chilling prior to the completion of the rigor mortis process (i.e., causing cold–shortening) [35]. Sometimes there can be problems on the opposite side where the meat is too soft or has low binding because of processing young animals (e.g., thin slices of oven-roasted turkey breast meat that are falling apart at the deli counter). This is not including the occurrence of myopathies such as the spaghetti meat syndrome, mentioned earlier, which results in muscle fiber separation in some young fast-growing broilers (Figure 5). Cases with tough breast fillet meat in young broilers (the so-called wooden breast syndrome) are also appearing today, where areas in the *Pectoralis major* muscle show degradation, necrosis and later the accumulation of connective tissue fibers and fat [36]. The occurrence of such myopathies in young fast-growing broilers in Canada can be very significant (Figure 6). Therefore, today all the players in the supply chain (i.e., breeders, nutritionists, farmers, meat processors) are working on ways to eliminate/reduce these myopathies. Reducing these myopathies is also very important to the primary meat processors who value a high degree of uniformity and homogenous muscle texture, especially when employing automated deboning equipment (see additional discussion below).

### 3.4. Juiciness

This part refers mainly to the consumers’ perception of juiciness, which is affected by the type of meat (dark vs. white broiler meat; the latter has more protein and less fat) and can also depend on primary processing procedures (e.g., water chilling vs. air chilling), storage time and conditions, cooking method and preparation. Overall higher moisture and/or fat level increases the perception of juiciness. For example, lean chicken breast fillet (without skin) contains on average 74% moisture, 22% protein and about 2% fat, while chicken thigh meat has 72%, 18% and 8%, respectively [38,39]. This by itself makes chicken leg meat perceived to be juicier when both types of meat are cooked in a similar way. Cooking chicken breast fillets in dry heat (oven, BBQ) without any marination will result in a pretty dry and chewy product. Therefore, quite a few convenient fresh poultry breast meat products are sold as marinated products (water and spices added; this information must be stated on the label). The method of chilling (i.e., water vs. air chilled) can also influence juiciness after cooking because water-chilled birds can be sold with some additional water incorporated during the process (about 6% for a medium size bird; value also depends on the bird size; see regulations for each specific country on the web). The water-chilled birds usually have more moisture at the beginning of the cooking cycle and depending on the amount of water retained (some can already come out during storage; called drip loss), cooking method and time, the final product can be perceived as juicier compared to air-chilled birds. However, since the processor has no control over the storage conditions of the meat after it leaves the plant, this is not a feasible way to enhance juiciness [40].

Moisture loss during processing (e.g., cutting), storage and distribution are commonly referred to as purge loss or drip loss. Processors and consumers try to minimize this amount as this is considered to be very expensive water. In cases such as PSE meat the level is obviously higher than in normal meat and values can reach 10–15%. This will obviously translate to the juiciness of the product. Unless the product is marinated or injected with a brine solution (water, salt/phosphate, and spices), the final cooked product will be very dry. This is not unique to poultry meat, and is actually seen in all meats (beef, pork, fish). As indicated before, lean muscle contains about 3/4 water, ‘water management’ is of great importance. Furthermore, during cooking meat proteins are denaturated and water is expelled from the meat structure due to both meat shrinkage (less space for water) and lower capacity of the desaturated meat proteins to bind water. This is better seen when dry heat (e.g., conventional oven) is used. Practical solutions for this include marination of whole muscle meat cuts [41] and straight water addition to ground sausage meat batters [13,42] where the added moisture can compensate for the losses during cooking. The ability of meat to retain moisture, with or without marination, also depends on factors such as genetics, body weight, ratio of lean meat to fat, stressors during catching and transportation of live animals to the plant, as well as chilling method (water vs. air as mentioned above) and freezing methods where slow freezing results in large ice crystal formation and more damage to muscle cells compare to fast freezing or individual fast freezing also known as IQF [43,44]. Another approach is to cook meat in liquid (e.g., soup, gravy) and count on the liquids surrounding the meat cut/portion. Examples of comprehensive reviews detailing the mechanisms of water holding capacity in meat and their relations to juiciness and the eating quality of meat are by Huff-Lonergan and Lonergan [44] and Warner [45].

### 3.5. Flavor and Aroma

Both flavor and aroma are developed during the heating process. This is due to chemical reactions among different components (proteins, fat, minerals, spices in the meat) that break down and later some of their smaller fragments become volatile and react with other small molecules induced by the elevated cooking temperature reviewed in [46]. This volatilization can also be influenced by diet and dietary supplements (e.g., fish oils, microalgae, vitamins) which affect the lipid content and composition of the meat.

In terms of meat processing, storage time and conditions can impact flavor and aroma. This can be the result of the so-called meat aging where endogenous enzymes (e.g., proteolytic, lipolytic) are breaking different components within the muscle and cause tenderization as well as the formation of small molecules that later participate in aroma development. Microorganism activity also results in different enzymes being released and breaking down large molecules (e.g., proteases breaking down proteins that can result in putrefied odor, discoloration, etc.), Furthermore, certain bacteria can secrete polypeptides that will appear on the product as slime. Rancidity can also occur as a result of a chemical reaction between fatty acids and reactive oxygen. This kind of oxidation is accelerated by high temperature (cooking, frying) but also happens at room temperature, as well as during frozen storage. Haugen et al. [47] described oxidation development during meat storage at −20 °C and also at warmer frozen temperature where the rate of rancid odor development increased under warmer conditions and longer storage time.

## 4. Convenience

Convenience and the ability to purchase products at a reasonable price are very important factors influencing consumer purchasing decisions. Currently, poultry is raised all over the world and is readily available in most places. Price is definitely competitive compared to other meats, as raising one kg of poultry is less expensive than beef, and pork [48]. This is due to a better feed conversion rate, significantly shorter growing period, etc. Poultry meat also has no religious limitations and is acceptable in all societies. Over the last century, the poultry industry has done a very good job in marketing and introducing new products as well as cut up portions. A good example is moving turkey meat sales from a seasonal cycle (before main holidays such as Christmas and Thanksgiving) to all-year-round marketing. This was done through the introduction of cut-up (e.g., one drumstick, or two wings in a package) and the introduction of new products. These products can be semi-prepared food items (e.g., chicken kabobs) that can be directly put on a BBQ, or fully ready-to-eat cooked products such as sliced turkey ham, oven-roasted chicken breast, and chicken nuggets. This allowed the industry to grow and become more competitive, efficient, and profitable. It is interesting to note that products such as turkey pastrami, ham, and bacon did not exist 50 years ago and today they capture about a quarter of the processed meat market. Today, there are even exclusive shops/farms selling premium organic-grown turkeys for $150 apiece before major holidays. The same is true for broiler chickens and ducks that are produced in limited numbers for very special markets. The last example was given to show the diversity and segmentation that took place in the industry and the industry’s ability to cater to different segments of the population.

Overall, convenience is a key driver in furthering innovation and is highly valued by modern consumer lifestyles. Consumers expect to be less involved in the food preparation process and want options for foods that can be eaten anywhere at any time [49]. As mentioned earlier, consumers today are spending 1/6 of the time on food preparation than consumers 50 years ago [1]. Chicken fillets are typically perceived by consumers to be a convenient choice due to their versatility, ease of preparation, and fast cooking time [31]. This convenience is further enhanced by the multitude of pre-prepared sauces, marinades, and/or pre-cooked options available to consumers today. Furthermore, chicken fillets are perceived to have less waste compared to other chicken cuts or meat products, i.e., little fat to trim, bones, and skin [31]. Although purchasing a whole chicken may be more economical in terms of price per kg, consumers may perceive it as wasteful and believe they are not getting sufficient value for their money. In addition, we see more consumers preferring not to spend time cutting / deboning meat and some say up front that they do not like to touch raw meat. In fact, 80% of poultry was sold as whole birds in N. America and Europe in 1960 and today this was reduced to less than 10% [49]. Since convenience is typically facilitated through additional processing, there are additional costs associated with more convenient products (e.g., ready-to-eat meals). However, consumers are now more willing to pay for convenience even during economic hardships.

## 5. Stability

**Figure 7:**
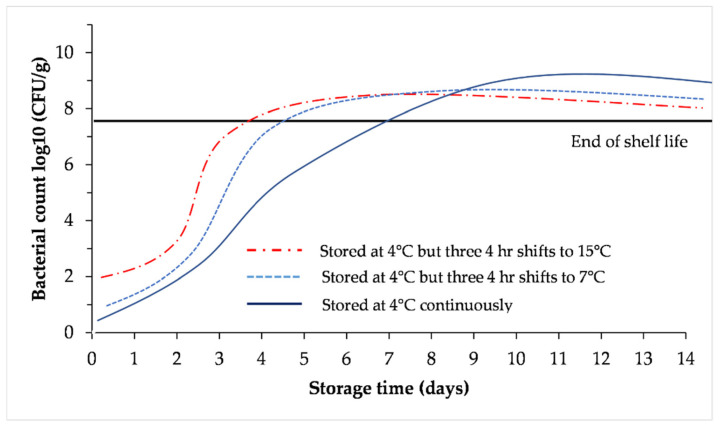
Growth of *Pseudomonas spp.* fitted with the Gompertz model on poultry during complete storage. Redrawn from Bruckner et al. [50].

It should be realized that the presence of microorganisms in/on the meat can result in both spoilage and the transfer of foodborne diseases such as *E. coli*, *Salmonella*, *Listeria*, and *Campylobacter*. This is a challenge since birds arriving at the plant carry microorganism on the outside (skin and feathers) and the inside (digestive and respiratory systems; 1 g of gut content can contain 10^8^ microorganisms). Bacteria use the meat components as an energy source for their growth and during the process also release different compounds such as enzymes and acids [50,51]. Viruses can also be carried out or transferred by the meat of sick/contaminated birds and avian influenza is an example. In a case of an avian influenza outbreak, the area/region is closed (by the government) and the birds are destroyed [52]. Figure 7 shows the stability of fresh poultry meat stored under different temperatures demonstrating increasing bacterial growth with higher temperatures and storage durations [50].

Shelf life is an important factor in consumer purchasing decisions because products with a longer shelf life are more convenient. Fresh poultry meat is perishable, like all other meats, as it has all the nutrients required for microorganism growth and does not have any inhibitory factors such as lysosomes in eggs, or low pH in citrus fruits. As many consumers prefer to buy fresh meat (as opposed to frozen) [53], the meat should be refrigerated or consumed right away. The latter is very common in places refrigeration/electricity is not available and/or where poultry is sold in so-called open or live markets. Recently governments in many jurisdictions started banning such markets as a result of the COVID pandemic [54,55]. In these markets, there are alternative ways to preserve meat for a longer period and they include the use of high levels of salt, drying (to reduce water activity), cooking to medium or high temperature (canning to produce shelf-stable products), and smoking (i.e., addition of antimicrobial compounds such as phenols and aldehydes). Radiation, both by an electron beam and isotopes such as cobalt, is also an available option, but special relative expensive facilities must be built and maintained [51].

**Figure 8:**
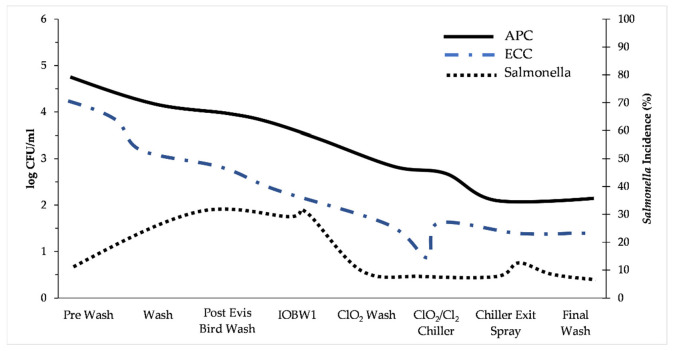
Microbial populations (log CFU per milliliter, mean SD) and Salmonella incidence (%) of carcasses following multiple interventions applied in sequence along the evisceration (slaughter) line at a poultry plant. The first point represents the stage before the first intervention, and every point thereafter represents the populations after the specific intervention. Post-Evis, post-evisceration wash; IOBW1, inside-outside bird wash 1ClO_2_, chlorine dioxide wash; ClO_2_-Cl_2_, chlorine dioxide wash plus chlorine chiller. Redrawn from Stopforth et al. [59].

So, in practice, the meat (poultry, beef, pork, fish) coming out of the plant is not sterile and has to be treated as such. The whole idea of the meat supply chain is to keep the number of bacteria coming out from the processing plant as low as possible so both the safety and the shelf life of the products will be ideal. The use of chemical intervention to reduce and control the number of microorganisms during processing is an important tool [56]. Common agents include chlorine, chlorine dioxide, peracetic acid, sodium hypochlorite, ozone, gamma irradiation, and trisodium phosphate, among others [57]. It should be mentioned again that differences between countries/regions exist, and for example, the use of antimicrobial rinses/dips is allowed in North America but not in Europe (note this is a big difference in philosophy between these two big trade regions). In any case, these chemical agents can be applied at different stages during primary processing including the inside-outside bird wash and chilling [16]. The overall goal is to reduce the number of microorganisms after each processing step. Figure 8 shows how such an approach can work by applying chlorine dioxide at strategic points during the process. However, the efficacy of these agents can be highly variable depending on the type of biocide, variant of pathogen, amount of organic material in the rinse water, and type of poultry being assessed [51,56]. Furthermore, contaminated retail poultry meat products can still occur (e.g., 22% of retail poultry products contaminated with *Salmonella* in the USA) which raised concern about the use of antimicrobial processing aids leading to antimicrobial resistance [56,58]. The interaction between biocide use and antimicrobial resistance in the poultry/red meat industry is a continuous research topic and investigations are underway into the use of alternatives to reduce the reliance on chemical decontaminants [56].

It is important to highlight the fact that the poultry industry managed to achieve significant success in reducing the number of microorganisms on fresh poultry meat. This reduction has been achieved by programs such as the base-line-monitoring program, where governments require plants to submit bacteria count results. The results are published (without identifying a specific plant) and serve as a moving standard for the industry to improve [60]. Plants that have bacteria counts above the standard (e.g., Salmonella positive samples) are inspected more often and their HACCP program is reviewed more frequently until they reach the standard.

Additionally, modern packaging of processed meat products (cooked, ready-to-eat) is designed to enhance stability by providing an oxygen barrier to slow down spoiling [49] as well as protect against lipid oxidation. This barrier is enhanced through vacuum or modified atmosphere packaging. Most processed poultry products today are sold in this kind of packaging; while there are benefits for stability, this again forces the consumer to rely on visual appearance of the product to guide purchasing decisions (discussed in more detail above). Modern packaging interplays with convenience by offering, e.g., resealable, microwave safe, family size/single serving options, room for a cooking suggestions panel. Aside from modern packaging, many techniques exist to enhance meat preservation and shelf-life including canning, drying, fermentation, salting, high-pressure processing, and smoking [49,61]. While these techniques are useful for enhancing shelf-life, there are impacts on flavor and texture which can be perceived favorably or unfavorably.

## 6. Wholesomeness

Food safety is a non-negotiable factor for all stakeholders due to the connections between foodborne illness and public health. Consumption of contaminated meat can cause human disease outbreaks from zoonotic pathogens (i.e., *Salmonella, Campylobacter* spp., *Listeria*) [56]. Managing these microbiological hazards is of the utmost importance for poultry/red meat processors. This is also the role of government regulations and inspection (enforcement of the law) at all food processing plants, including meat plants. Each country or a trade group (e.g., the European Union) has a set of detailed regulations that covers many aspects, starting with a list of forbidden feed additives to the inspection of live birds prior to slaughter, during processing and later during storage and distribution. These regulations (can be found on the internet) typically contain a few hundred pages and attempt to cover all areas of the supply chain. Today, most countries require food processing plants to use a hazard analysis critical control (HACCP) program to help streamline the process and place some of the responsibilities on the manufacturing industry. Each plant needs to have a certificate to operate. These certificates also include approval of the plants’ prerequisite requirements (need to satisfy regulations about items such as the building materials, use of nontoxic paint on walls in the processing area, pest control program, employee training), and the HACCP program itself that covers all the steps during the actual production of the product (e.g., chilling the meat, washing contamination, cooking products). It should also be mentioned that quite a lot of variation can exist among countries. For example, some countries require the inspection of the whole live flock prior to starting primary processing, while others require the inspection of each individual bird after bleeding and evisceration. The overall idea is the same, and it is to prevent the entry of sick/deceased/contaminated animals that can transfer zoonotic diseases (e.g., *Campylobacter*) to people, into the food chain. The pathogens mentioned above can be those affecting humans but not necessarily the birds; i.e., birds are just a healthy carrier of some Salmonella serotypes (not all the 2500) that are dangerous to people. As indicated above, regulations among countries can vary, however, if one wants to export meat to another country they need to comply with that country’s regulations.

Traceability is also becoming very important today both in terms of regulatory requirements and as a marketing tool. Data can be recorded at all stages of processing and production which can be used to connect poultry products (end-point) with birds on the farm and at processing [62]. In terms of regulation, it is critical to be able to find the source of a problem (e.g., microbial, chemical). An example of a chemical contamination problem is dioxin which ‘appeared’ in European poultry meat and eggs in 1999. This caused the large-scale destruction of millions of eggs and poultry carcasses that were exposed to dioxin that ended up in poultry feed [63]. Typically, multiple assurances of traceability (e.g., on-farm for feed and welfare, at the processing plant for food safety) are more highly valued than a single traceability measure [64]. The meta-analysis of Cicia and Colantuoni [64] showed that consumers are willing to pay 22% more over the base price for a meat product that displays multiple traceability attributes (i.e., on-farm traceability, country of origin), and they are also willing to pay 167% more over the base price for a product that displays on-farm traceability (i.e., the meat’s “on-farm production path”).

## 7. Nutritional Value

Poultry meat, like other meats, is a good source of proteins (regular and essential amino acids), vitamins, and fat [38]. Fat composition is unique as poultry has more unsaturated fatty acids compared to red meat, therefore it is considered by many consumers as a healthier meat source. It also has less fat in certain cuts. For example, skinless chicken breast fillet has only 2.5% fat and no marbling. Most fat is present under the skin (subcutaneous fat), meaning that skinless fillet has very little fat; mainly polyunsaturated fatty acids present in cell membranes. Skinless broiler drum meat typically has 6% fat and with skin about 10% fat [38]. Poultry breast meat is a poor source of iron (about 0.37 mg/100 g raw or 0.49 mg/100 g cooked [65]), and that can be seen by the very light color of the meat (low myoglobin content). However, raw duck breast meat has about 4.51 mg/100 g myoglobin and appears dark red [66]. The difference is because of the high concentration of red muscle fibers, designed for prolonged flights in these migratory-type birds.

The approximate caloric value of 100 g portions of cooked [38]:

- Skinless chicken breast fillet = 158 kcal
- Skin on chicken breast fillet = 197 kcal
- Skinless chicken thigh meat = 179 kcal
- Skin on chicken thigh meat = 226 kcal

For comparison, the approximate caloric value of 100 g portions of cooked

(See fdc.nal.usda.gov web site):

- Beef leg meat = 201 kcal
- Pork leg meat = 211 kcal
- Beef brisket = 244 kcal
- Pork chop = 255 kcal

In terms of meat further processing and meat product quality, one should remember that the higher proportion of unsaturated fat in poultry meat results in a lower melting point. This must be taken into account when preparing emulsified meat products such as chicken/turkey frankfurters and bologna. In those cases, chopping end-point temperature must be kept low (8 °C) than when red meat products are made (12–15 °C) to prevent fat separation during the later cooking operation [67].

Overall, poultry products are typically perceived as lean, low-fat foods that are healthier than other red meat products [31,68]. An example is turkey bacon which commonly has about half the fat and salt content of pork bacon. This is because turkey bacon is prepared from finely chopped white breast meat and layers of finely chopped (or intact) dark leg meat. This is again an example of how poultry meat can be marketed. It is interesting to note that in this particular case, using the name turkey bacon caused initial lawsuits from the pork industry, trying to block the poultry industry from using such a name. Today, further strides are being made in improving the healthiness of processed poultry products; in particular, offering products with reduced salt, nitrites, and fat [69]. For example, the sodium concentration in chicken nuggets (i.e., sodium chloride required for extracting the salt-soluble proteins from the meat to facilitate binding/gelation during cooking) can be reduced to some extent by partially replacing with potassium/calcium chloride without affecting consumer acceptance [70].

## 8. Ethical Aspects

Earlier qualitative studies indicated that consumers of poultry products preferred to be disconnected from the animal’s origin and preferred not to think about how the animals were raised [31]. This was related, in part, to consumers’ preference for cut-up chicken portions instead of whole birds to disconnect further from the animal. While the preference for cut-up portions persists (likely due to convenience discussed above), consumers today show an increasing desire to know more about where their food came from and how it was raised.

Many alternative production strategies (e.g., free-range, organic, slow-growing, anti-biotic free) have evolved over the years as consumer expectations for animal health & welfare and product traceability change. Products from alternative systems/strategies are typically perceived by consumers as more natural, superior in quality, less risky in terms of foodborne illness, and healthier compared to conventionally produced products. An example where people are interested in buying slow-growing breeds that have been raised for >80 days and are considered to be more flavorful and have a better texture is France. The Label Rouge brand is premium type poultry where consumers are willing to pay more. In fact, 60% of the whole bird market in France is the Label Rouge brand, while 13% is Certified, 10% Organic and only 16% is conventionally raised broilers [71]. In the cut-up chicken segment, only 12% is from Label Rouge and 60% from conventionally raised birds. In the USA, for comparison, less than 10% of the whole bird market is from organic or slow-growing birds. In contrast, a survey of Polish consumers indicated that most respondents (56%) do not pay attention to the type of production system their poultry is coming from [53]. Of the 44% that do consider rearing system, more people were inclined to choose conventional (25%) over organic rearing (19%), although this is potentially due to the higher price of organic products [53]. Therefore, there may be geographical variation in consumer demand for alternative products which will need to be addressed by producers and processors. It’s also interesting to note that a recent survey of Italian consumers indicated that consumers found modern broilers to have better taste and texture compared to slow-growing traditional breeds [72]. This difference in acceptability was potentially due to the greater amount of haem iron (more metallic taste) and tougher texture in the slow-growing breeds compared to the modern breed). In summary, consumer preferences may change depending on their social, cultural, or geographical environment which adds another layer of complexity to developing poultry products.

While the adoption of alternative strategies may improve the social acceptability of the poultry industry, there may be production limitations. Slow-growing breeds require a longer production period which increases the number of resources required and wastes produced [73]. Additionally, longer production periods increase the risk of exposure of poultry to diseases and environmental contaminants which could be present as chemical hazards in the meat [74]. As discussed above, carcass bruises and discolorations can be problematic for processors. Broilers raised in a free-range environment have been reported to have more bruises, typically on the breast, compared to those raised in extensive indoor systems [26].

Due to the greater resources required to raise alternative products, prices must be higher; however, consumers are not always willing to pay the price [73,75]. Willingness to pay for alternative products is influenced by consumer demographics (i.e., consumer age, income), package labelling/branding, and personal value of price, taste, and animal welfare [76]. Willingness to pay tends to decrease as the base price of the product increases which may pose a problem for products from alternative systems with typically higher base prices [64].

## 9. Conclusions

Poultry processors must continually evolve to keep pace with the demands of the modern consumer. Over the past few decades, the poultry market has changed considerably with most products being sold as cut-up or further processed products. This review focused on challenges faced by poultry processors in the different areas of product quality: eating quality (i.e., color, texture, flavor, juiciness), convenience, stability, wholesomeness, nutritive value, and ethical aspects. In brief, the increased automation and mechanization of poultry processing hinges on a high level of bird uniformity and a low level of defects (i.e., myopathies) both of which are becoming increasingly problematic. Moreover, consumers also expect uniformity in terms of product weight, color, absence of broken bones/bruises as well as convenient and safe products. Additionally, consumers today place greater value on a product’s ethical aspects and are seeking out organic, free-range, slow-growing, or antibiotic-free poultry products. Although these products may have benefits for animal welfare and social acceptability, they come with their own challenges such as more resources required, possibility of more defects, and higher costs for the consumer. Addressing these challenges will rely on solutions developed collaboratively with all stakeholders in the poultry industry including breeders, farmers, and processors.

## Data Availability

Not applicable.
